# EHMTI-0035. "Cervical pain" study in an Italian tertiary referral headache center

**DOI:** 10.1186/1129-2377-15-S1-D69

**Published:** 2014-09-18

**Authors:** M Viana, G Sances, G Nappi, G Sandrini, C Tassorelli

**Affiliations:** 1 Headache Science Center, C. Mondino National Neurological Institute, Pavia, Italy

## Introduction

The majority of migraine patients remain undiagnosed or misdiagnosed in Italy (Cevoli et al. 2009). In our experience, many patients affected by migraine self-diagnose it as 'cervical pain syndrome' (CP) assuming cervical spine pathology as the cause.

## Aim

To phenotype and classify, in a tertiary referral headache center, the headache types of patients with self-diagnosed CP, and to describe this sample of patients.

## Methods

All patients aged 18 to 75yo, referred to Mondino Headache Center for a first visit for headache, completed a questionnaire about CP. A detailed history was taken and a neurological exam was performed in each patient. Brain and cervical imaging were performed when deemed necessary. All patients finally received a diagnosis based on ICHD-IIIβ criteria.

## Result

85 patients completed the questionnaire: 47 were suffering from self-diagnosed CP, 3 had suffered from self-diagnosed CP, 35 never had self-diagnosed CP. In all of 50 CP descriptions, the pain involved the head. ICHD-IIIβ diagnoses included migraine without aura (n=30), migraine with aura (1), probable migraine without aura (n=4), chronic migraine (n=7), medication overuse headache/chronic migraine (n=5), tension type headache (n=2), hemicrania continua (n=1), no patient presented with a phenotype suggestive of cervicogenic headache. 24 out of 50 patients with CP answered the question 'who did tell you that these attacks are CP?' with: general practitioner/medical specialist. The majority of these patients underwent exams without a clear indication and ineffective treatment.

## Conclusion

To the best of our knowledge, this is the first study that systemically assessed the headache phenotype of patients with self-diagnosed CP. The results suggest that the phenomenon of self-diagnosing CP is very common in Italy, even in patients referred to a tertiary headache center. The majority of these patients suffer from typical migraine attacks, without any evidence of pathological conditions of the cervical spine.

No conflict of interest.

